# Horizontal Gene Transfer as a Source of Conflict and Cooperation in Prokaryotes

**DOI:** 10.3389/fmicb.2020.01569

**Published:** 2020-07-17

**Authors:** Rebecca J. Hall, Fiona J. Whelan, James O. McInerney, Yaqing Ou, Maria Rosa Domingo-Sananes

**Affiliations:** ^1^School of Life Sciences, University of Nottingham, Nottingham, United Kingdom; ^2^Division of Evolution and Genomic Sciences, School of Biological Sciences, Faculty of Biology, Medicine and Health, The University of Manchester, Manchester, United Kingdom

**Keywords:** horizontal gene transfer, conflict, cooperation, antimicrobial resistance, natural selection

## Abstract

Horizontal gene transfer (HGT) is one of the most important processes in prokaryote evolution. The sharing of DNA can spread neutral or beneficial genes, as well as genetic parasites across populations and communities, creating a large proportion of the variability acted on by natural selection. Here, we highlight the role of HGT in enhancing the opportunities for conflict and cooperation within and between prokaryote genomes. We discuss how horizontally acquired genes can cooperate or conflict both with each other and with a recipient genome, resulting in signature patterns of gene co-occurrence, avoidance, and dependence. We then describe how interactions involving horizontally transferred genes may influence cooperation and conflict at higher levels (populations, communities, and symbioses). Finally, we consider the benefits and drawbacks of HGT for prokaryotes and its fundamental role in understanding conflict and cooperation from the gene-gene to the microbiome level.

## Introduction

Organisms typically transmit genetic information vertically to their offspring, but occasionally DNA is acquired horizontally from other sources. This horizontal gene transfer (HGT) is particularly prevalent in prokaryotes, where it is one of the main mechanisms contributing to genetic variation and thus evolution. HGT can occur by transformation (the cellular uptake of exogenous DNA; Griffith, 1928), transduction (the movement of chromosomal DNA *via* viruses; Zinder and Lederberg, 1952), and conjugation (DNA transfer *via* cell-cell contact; Lederberg and Tatum, 1946), as well as other mechanisms (García-Aljaro et al., 2017). HGT is not entirely random, however, and there are several barriers to genetic transfer. Exogenous DNA must first enter the cell, evading enzymatic cleavage (Thomas and Nielsen, 2005), and then be stably maintained by incorporation into the genome, either through homologous or illegitimate recombination or by continued replication on extrachromosomal elements (Gogarten and Townsend, 2005). Successful HGT is therefore unlikely, with only a fraction of internalized DNA maintained through vertical transmission in the long term (Thomas and Nielsen, 2005).

Most acquired DNA is likely to be neutral or deleterious, a large component of the latter in the form of selfish mobile genetic elements (MGEs) including transposons, integrated prophages, and integrated or self-replicating plasmids (Vos et al., 2015). Beneficial traits that promote adaptation to new environments can also be acquired by HGT, notably genes involved in metabolism and antibiotic resistance (Gogarten and Townsend, 2005; Baltrus, 2013; Polz et al., 2013; McInerney et al., 2017). Transferred genes may or may not share the interests of the recipient genome, depending on their fitness effects. Here, we define interest as maximizing fitness of each interacting partner (e.g., the incoming gene and the host cell). This is similar to classical definitions of cooperation and conflict in social environments in which individuals are “agents” that interact with each other. These agents represent the level at which natural selection acts and adaptation takes place and can range from single genes to groups of organisms (Foster, 2011).

**Figure 1:**
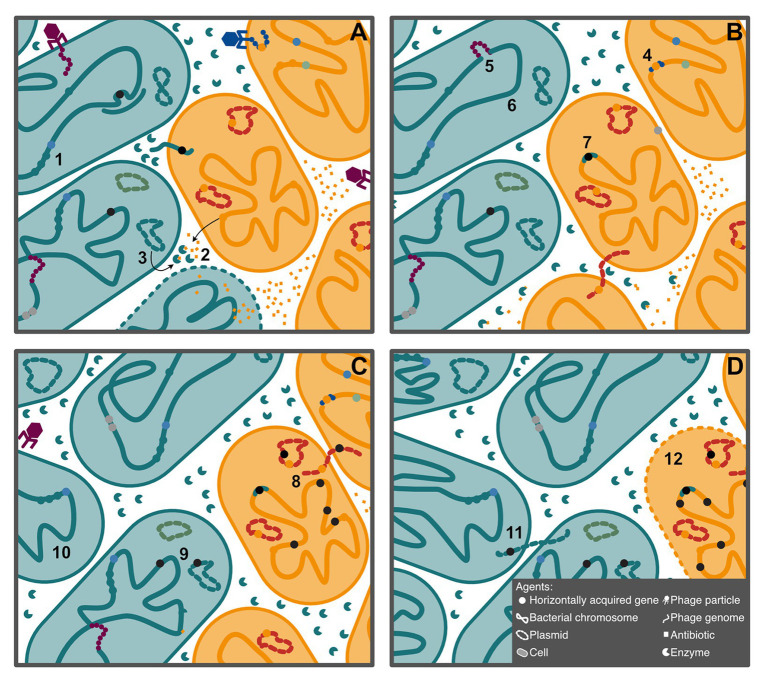
Schematic description of the agents considered in this article, with examples of some of their interactions. Shapes indicate the type of agent, while color represents the type of interaction. Agents of the same color cooperate, and agents of different colors conflict with one another. **(A)** Acquisition of three genes allows the teal bacteria to colonize a niche dominated by the yellow bacteria (1). The yellow bacteria, however, produce an antibiotic capable of killing the teal cells (2). In this novel environment, teal bacteria have acquired a plasmid encoding an antibiotic-degrading enzyme (3), which allows them to expand their population. **(B)** The antibiotic producing gene can be acquired by transduction in new cells (4). Selfish phage can also insert in the chromosome (5). Transformation followed by homologous recombination can eliminate selfish elements from the chromosome (6) but can also lead to gain of selfish elements (7), in this case by illegitimate recombination. **(C)** In some backgrounds, these selfish elements can expand, increasing the chance of insertion in essential parts of the genome (8). These elements can also insert in conjugative plasmids, which can then be transmitted to other cells (8 and 9). Among the population of teal cells, cheats arise that do not produce the antibiotic degrading enzyme (10). **(D)** Reacquisition of the enzyme encoding plasmid by conjugation restores cooperation, although it also introduces the selfish element (11). The expansion of selfish elements results in the death of the yellow cell (12), as the teal population continues to expand.

Here, we consider genes, plasmids, phage, genomes, and organisms as agents that interact on multiple levels (Figure 1). These agents cooperate when they are mutually beneficial, that is, when they have the same interests, and each agent enhances the fitness of the others, perhaps by facilitating the emergence of diverse, novel phenotypes or interactions, such as a symbiotic relationship. An acquired gene may, for example, increase the fitness of a cell, while benefiting from vertical transmission. Conflict occurs when the interests of the agents differ. A conjugative plasmid may, for example, not provide any benefit to the recipient cell, while exploiting resources to enhance its horizontal and vertical transmission. In this perspective article, we consider situations in which HGT contributes to driving conflict and cooperation within and between genomes, and we discuss why HGT is so pervasive given its risky nature.

## Conflict and Cooperation Within Genomes

First, we consider interactions between single horizontally acquired genes (HAGs) and the recipient cell. We expect that a certain cohort of HAGs will have a neutral fitness effect on the recipient cell, with their frequency in the population consequently being determined purely by drift (Foster, 2011; Knöppel et al., 2014; Andreani et al., 2017; Bobay and Ochman, 2018). Other HAGs will be deleterious, and therefore in conflict with the genome. Often, this is because transferred genes are selfish elements that increase their own fitness, usually through further horizontal transmission, at the expense of the host cell. Selfish elements with mild or strong deleterious effects will be purged from the population by selection (Scott and West, 2019). When gained at a high enough rate, selfish HAGs may, however, be maintained through constant HGT, becoming chronic genomic “infections” (Iranzo et al., 2016; Domingo-Sananes and McInerney, 2019). This HAG-genome conflict would lead to the evolution of mechanisms that prevent these genomic parasites, as discussed in the “If HGT Is Risky, Why Do It?” section. Other genes may be deleterious due to incompatibility with the recipient cell and are likely lost due to selection. In this instance, all interacting agents lose both the acquired gene and all the other genes in the genome.

Some HAGs are undoubtedly beneficial to the recipient cell. We regard this type of interaction as mutual cooperation, with fitness gains for the cell, all its resident genes, and the newly acquired gene, the latter of which can now spread in a new population. These beneficial genes can allow the colonization of novel niches or facilitate survival in changing environments, as shown by the spread of antimicrobial resistance (AMR) genes (Von Wintersdorff et al., 2016). Cooperative interactions can also be observed between multiple HAGs within the same cell, particularly if they have minimal or deleterious effects on cell fitness as individuals but confer a benefit together. This is illustrated in multi-protein complexes or pathways where each protein relies on others to realize their combined function. Often, such groups of genes conferring a function may be found as operons, clustered in a single stretch of DNA. It has been proposed that this facilitates the transmission of the group of genes to other cells and allows them to act as an independent agent, a selfish operon (Lawrence, 1999).

Groups of HAGs can lead to adaptation to novel environments, even resulting in ecological divides between organisms with shared ancestry. Thus, HGT can start the process of diversification between linages that can then co-exist indefinitely as proposed by the ecotype model (Wiedenbeck and Cohan, 2011; Lassalle et al., 2015; Cohan, 2017). A striking example is the evolution of halophilic archaea. Phylogenetic analyses consistently place the halophilic archaea within a methanogen clade (Brochier-Armanet et al., 2008; Makarova et al., 2010; Kelly et al., 2011), even though these two groups usually occupy very different ecological niches. The transition of halophiles from within these methanogens was driven by the acquisition of 1,089 genes (Nelson-Sathi et al., 2012). This likely created barriers between the halophiles and methanogens to further HGT; genes that are required to be a methanogen are not observed in halophile genomes and vice versa (Rhodes et al., 2011). Diversification of populations due to HGT can, however, be slow if homologous recombination between prokaryote groups continues outside the adaptive HAGs, as proposed by the fragmented speciation model (Retchless and Lawrence, 2007, 2010).

HAGs can also conflict with one another. A pair of genes may be beneficial individually but become harmful together, if, for example, the product of *geneA* is detrimental to the product of *geneB*. It may also be harmful to the fitness of the cell to maintain multiple genes with redundant functions. This is possibly at play in the siderophore families of *Salinispora* spp., where only one of the two possible families (desferrioxamine or salinichelin) is ever observed in any given strain despite frequent HGT of the encoding gene clusters (Bruns et al., 2018). It is likely that these gene clusters do not appear together because of functional redundancy, given that both siderophores are functionally nearly identical. Selection at the level of interactions between organisms may also be of influence here, as discussed in the “Conflict and Cooperation Between Genomes” section. Groups of HAGs may conflict directly with the interests of the cell to enhance their own transmission; an inserted prophage, encoding multiple genes required for self-construction, can kill the cell upon entry into the lytic phase. Overall, interactions between HAGs should create distinctive patterns of association or correlation when their presence or absence is analyzed across large groups of related genomes. Cooperation between HAGs could lead to statistically significant gene co-occurrence, conflict can result in gene-gene avoidance (or anticorrelation), and dependencies can create conditional relationships within genomes (Cohen et al., 2012, 2013; Behdenna et al., 2016; Press et al., 2016; Pensar et al., 2019; Whelan et al., 2020).

This perspective poses several questions about the interactions occurring in prokaryotic genomes as a result of HGT. First, in instances where groups of HAGs function together, it is interesting to question whether these genes are acquired simultaneously or sequentially. It is reasonable to hypothesize that the acquisition of one gene may in some instances serve as a gateway to gaining another. This could happen if *geneA* is beneficial and *geneB* deleterious on their own, but both genes together become highly beneficial for the cell, resulting in the presence of *geneB* being conditional on the presence of *geneA*. These types of patterns have been observed upon analysis of hundreds of bacterial genomes (Press et al., 2016). Another possibility is that gain of an “event horizon” gene could mark the start of a transition to a new environment (McInerney et al., 2020). Second, the prevalence and magnitude of variations in fitness contributions of genes in different environments and through time are not known. Some AMR-associated genes are, for example, beneficial in the presence of the antibiotic but deleterious in its absence because of the associated fitness cost (Baltrus, 2013; Hernando-Amado et al., 2017; San Millan and MacLean, 2017). It is not yet known how common these spatial and temporal fitness effects are. Finally, an understanding of the degree to which the recipient’s genetic background can affect the fitness contribution of a HAG to the cell is important in establishing the potential for cooperation and conflict. Together, these considerations affect the propensity for conflict and cooperation between HAGs and the recipient genomes, with the degree of impact requiring further study.

## Conflict and Cooperation Between Genomes

Genomes, cells, and organisms (of the same or different species) can also be considered as interacting agents. Many examples of these types of interactions exist, including cooperation (e.g., production of common goods) and conflict (e.g., antibiotic and bacteriocin production). These social and ecological interactions between prokaryotes are widespread and highly prevalent in contexts where multiple cells have the opportunity to interact, such as in biofilms, microbiomes, and symbioses (West et al., 2006; Foster, 2011). Here, we focus specifically on how HGT can play major roles in shaping such interactions.

Production of public goods is one of the main forms of cooperation between prokaryotes and includes the secretion of products required to build biofilms, digest complex chemicals, and modulate the immune response of a host, among other important functions (West et al., 2006; Rakoff-Nahoum et al., 2016; Lerner et al., 2017). Genes involved in public good production can be transferred between organisms, creating novel opportunities for cooperation and adaptation (Mc Ginty et al., 2011; Rankin et al., 2011). In *Escherichia*, for example, most secreted proteins are of recent origin, likely acquired through HGT (Nogueira et al., 2009). A large number of secreted proteins are thought to be public goods and to contribute to interactions between cells. While these goods are normally beneficial to the producing cell, non-producers can take advantage without paying the associated costs of production. This may still increase the producer’s inclusive fitness (the capacity of an individual to produce descendants plus its effect on the reproduction of other individuals weighted by relatedness; West et al., 2007) in situations with limited dispersal, where the benefits are likely to be received by close relatives. Furthermore, constant HGT of public good genes can promote cooperation, at least in structured populations, by allowing the re-acquisition of public good production in cheats (Mc Ginty et al., 2011; Dimitriu et al., 2014). Cooperation may be further stabilized by linking production of public goods to recognition of relatives. This is one of the possible explanations for the existence of quorum sensing and other kin-recognition systems. An interesting case is the production of strain-specific siderophores that can only be used by close relatives (West et al., 2006). On the other hand, kin-recognition systems can also be horizontally transferred, creating more opportunities for cooperation and conflict between non-relatives. Conflicts in kin recognition may have led to the existence of complex, combinatorial recognition systems, such as those observed in *Bacillus subtilis* (Lyons et al., 2016).

At a higher level, we can consider interactions between members of a microbiome and their host. In particular, we focus on the well-described interactions between symbiotic prokaryotes and their host. HGT often plays a role in maintaining the cooperative nature of symbioses. Syntrophy, where metabolites produced by one partner are consumed by the others, is a clear example of between-organism cooperation. In the symbioses, where one organism physically resides within a host, the latter may exhibit cooperative behavior by not eliminating the former, allowing the relationship to establish and persist (Chung et al., 2018). There is, however, always the potential for conflict; the provision of a benefit is almost always costly for the symbiont, possibly allowing cheats to arise within the symbiont population (Douglas, 2008). The presence of cheats could result in conflict between members of the bacterial population or between host and microbiome.

In symbiosis, the sharing of metabolic intermediates can lead to collaboration between cells (of the same or different species), while potentially reducing genome size (Morris et al., 2012; Fullmer et al., 2015). HGT can have an important role in establishing these relationships. A well-characterized example is *Buchnera aphidicola*, the obligate bacterial symbiont of the aphid *Acyrthosiphon pisum*. Several genes essential to *Buchnera* are encoded in the *A. pisum* genome, having been previously transferred from a different bacterial species (Shigenobu et al., 2000; Nikoh and Nakabachi, 2009; Nikoh et al., 2010). An even more intricate example is the cooperative relationship between the mealybug *Planococcus citri* and its microbiome. *P. citri*’s symbiont *Tremblaya princeps* has a drastically reduced genome, possibly facilitated by the acquisition of its own bacterial symbiont, *Moranella endobia* (McCutcheon and Von Dohlen, 2011). At least 22 expressed genes of bacterial origin, that came from neither *Tremblaya* nor *Moranella*, have been identified in the *P. citri* genome (Husnik et al., 2013) and, in some instances, complement genes that have been lost by the mealybug’s microbiota. Several peptidoglycan-related genes are expressed by the host and are thought to work together with genes retained by *Moranella* to control the integrity of its cell wall (Bublitz et al., 2019). The insect likely has no need for these genes besides maintaining its bacterial microbiome, demonstrating cooperative interactions between host and symbionts.

These interactions raise important questions on the nature of symbioses and microbiomes, namely, at what point does a symbiont become obligate, and how is this driven by HGT? Has ancient HGT from bacteria to host enabled these symbionts to become more of an organelle than otherwise would have been possible? How does HGT between members of the microbiome affect its interaction with the host? It could be argued that these symbiotic interactions go beyond mere cooperation. The preservation by the host of genes lost by its symbionts may be random but might also demonstrate functional integration and unity between host and microbiome. In these examples, HGT has allowed organisms to become inter-dependent, ensuring cooperation in maintaining their relationship. From a wider perspective, many questions remain regarding the degree of influence that HGT exerts on interactions between organisms. The fitness effects of HAGs in different cells and environments should be considered, as should their effects in social contexts.

## If HGT Is Risky, Why Do It?

Given the prevalence of genetic parasites and the high probability of acquiring deleterious, or at best neutral genes, it is worth considering why cells engage in HGT despite its risky nature. It is possible that the cell does not have a chance to weigh the risks and instead is constantly battling deleterious incoming DNA against which they cannot always adequately defend (i.e., passive HGT). Another possibility is that HGT occurs because even though transferred genes are deleterious overall, their average fitness effects are not high enough for selection to either eliminate them or to lower HGT rates further (drift-barrier situation; Sung et al., 2012). These situations are likely in the case of transduction and conjugation, where viruses and plasmids usually act as selfish agents. The role of transformation may be harder to explain in this framework, although it has been proposed that the main role of transformation is the elimination of integrated, harmful DNA, in particular transposons (Croucher et al., 2016).

Consistent with the potential dangers of HGT, prokaryotes possess mechanisms to block it. These systems can be innate [restriction modification (RM)] or adaptive [clustered regularly interspaced short palindromic repeats (CRISPR)-Cas], and are present in most prokaryote genomes (Horvath and Barrangou, 2010; Oliveira et al., 2014; van Houte et al., 2016). These systems are thought as mechanisms that inhibit HGT but they can also promote it, with transduction enhanced by the presence of certain CRISPR-associated spacers (Watson et al., 2018; Varble et al., 2019). Furthermore, RM and CRISPR-Cas systems have been found within mobile genetic elements (Oliveira et al., 2014; Faure et al., 2019), indicating that they themselves can be transferred between organisms, creating the potential for even more complexity.

The existence of cell-encoded mechanisms that promote HGT indicates that HGT may overall be more beneficial than harmful. As discussed, HAGs can help cells adapt to novel environments, and the benefits of homologous recombination to remove deleterious DNA and reduce clonal interference may help to maintain HGT (Croucher et al., 2016; Iranzo et al., 2016; Rocha, 2016). The presence of mechanisms that promote and prevent HGT imply that second-order selection could act on the rates of gene gain and loss, leading to diversity in the evolvability of prokaryote genomes in different clades (Vos et al., 2015). The capacity for HGT may, for example, decrease (e.g., diminished competence or increased defense) in niche-restricted organisms, in which the potential benefit of HGT is low. There is indeed variation in the rates of gene gain in different prokaryotes (Puigbo et al., 2014), but the diversity in transfer rates for different genes and taxa, and to what extent a selection can act on them, is not yet known.

Cells also have mechanisms that regulate the rates of HGT at different points in their life cycle, and evolution may act on these mechanisms to maximize benefits and minimize costs. In *Streptococcus pneumoniae* and *Streptococcus mutans*, quorum sensing regulates competence, potentially promoting genetic exchange between members of the same species, resulting in both the removal of deleterious mutations or genes and acquisition of beneficial ones (Shanker and Federle, 2017). Regulation of HGT during the lifetime of a population may also lead to the evolution of bet-hedging strategies, where only a fraction of a population of closely related individuals engages in HGT (Veening et al., 2008; Polz et al., 2013; Croucher et al., 2016).

Constrained genome sizes and high rates of gene loss, coupled with pressures in maintaining multiple pathways and functions, may make HGT beneficial in prokaryotes. A phenotype (e.g., production of public goods) could be maintained collectively by a group of cells that each encode different genes required for that phenotype (Morris et al., 2012; Fullmer et al., 2015). Constraints in the extent to which prokaryotes can plastically respond to the environment through regulation of gene expression and protein activity may also play a role. This is due to the tendency of the number of regulatory genes to scale quadratically with genome size, whereas genes belonging to most other functional categories in the genome scale linearly or remain constant (Molina and van Nimwegen, 2009). HGT could, therefore, allow the acquisition of ecologically restricted genes that would not be maintained outside the niches, where they confer a fitness benefit, due to high regulatory costs. These possibilities bring further questions, including whether genome size in prokaryotes is constrained by selection or high rates of gene loss, and under which circumstances can the sharing of functions be evolutionary stable.

Acquiring new genes will always carry the risk of deleterious or neutral effects, but the benefits of colonizing novel niches may outweigh the risk. Despite progress in understanding many of these hypotheses, we still do not know the main reason why HGT is so prevalent, and a single explanation is unlikely. As more genomes become available, analysis of many genomes from the same and multiple species will help tackle some of these questions.

## Concluding Remarks

We are only just beginning to understand the multifarious nature of the drivers of genome composition. We have several known unknowns; the rate of gene acquisition by HGT, the distribution of fitness effects that are mediated by incoming genes, the frequency with which innate and acquired prokaryotic defense systems are called into action in natural environments, and the magnitude of intragenomic conflicts, or indeed the strength of the effects of cooperation between recipient cells and HAGs. Furthermore, we do not know to what extent gene sharing and content variation affect conflict and cooperation between the prokaryote genomes and cells in which they reside. Understanding these interactions may also require clarifying at which level selection is acting, that is, clearly defining the agents, a difficult task (Foster, 2011).

The high levels of HGT seen in prokaryotes means that the study of these distinct cooperative and conflicting interactions is central to our understanding of prokaryotic evolution. Thanks to genomics, we can now easily acquire the “parts list” of genomes. The next step will involve our understanding of how they all fit together, and why.

## Author Contributions

RH, FW, JM, YO, and MD-S conceived of, drafted, and approved this manuscript. JM secured necessary funding to support the work of this manuscript. All authors contributed to the article and approved the submitted version.

### Conflict of Interest

The authors declare that the research was conducted in the absence of any commercial or financial relationships that could be construed as a potential conflict of interest.
